# Health Facilities Readiness and Determinants to Manage Cardiovascular Disease in Afghanistan, Bangladesh, and Nepal: Evidence from the National Service Provision Assessment Survey

**DOI:** 10.5334/gh.1311

**Published:** 2024-03-20

**Authors:** Md. Durrul Huda, Mosiur Rahman, Md. Golam Mostofa, Prosannajid Sarkar, Md. Jahirul Islam, Izzeldin Fadl Adam, Nguyen Huu Chau Duc, Saber Al-Sobaihi

**Affiliations:** 1 Diabetic Hospital, Chapai Nawabganj, Bangladesh; 2 Department of Population Science and Human Resource Development University of Rajshahi, Rajshahi-6205, Bangladesh; 3 Dr. Wazed Research and Training Institute, Begum Rokeya University, Rangpur, Bangladesh; 4 Griffith Criminology Institute, Griffith University, Mount Gravatt, QLD 4122, Australia; 5 Faculty of Public Health, University of Khartoum, Sudan; 6 Hue University of Medicine and Pharmacy, Hue University, Vietnam; 7 Premium Research Institute for Human Metaverse Medicine (WPI-PRIMe) at Osaka University, Osaka, Japan

**Keywords:** cardiovascular diseases, health facilities readiness, health services, service provision assessment survey, South Asia

## Abstract

**Background:**

In South Asia, cardiovascular diseases (CVDs) are an increasing public health concern. One strategy for dealing with the growing CVDs epidemic is to make health facilities more ready to provide CVDs services. The study’s objectives were to: (1) assess healthcare facilities’ readiness to offer CVDs services; and (2) identify the variables that influence such readiness.

**Methods:**

This study employed data from the Afghanistan Service Provision Assessment Survey 2018–2019, Bangladesh Health Facility Survey 2017, and Nepal Health Facility Survey 2021 that were cross-sectional and nationally representative. In Afghanistan, Bangladesh, and Nepal, 117, 368, and 1,381 health facilities, respectively, were examined. A total of 10 items/indicators were used to measure a health facility’s readiness to provide CVDs services across three domains.

**Results:**

The mean readiness scores of managing CVDs were 6.7, 5.6, and 4.6 in Afghanistan, Bangladesh, and Nepal, respectively. Availability of trained staff for CVD services are not commonly accessible in Afghanistan (21.5%), Bangladesh (15.3%), or Nepal (12.9%), except from supplies and equipment. Afghanistan has the highest levels of medicine and other commodity availability. Among the common factors linked with readiness scores, we ought to expect a 0.02 unit rise in readiness scores for three nations for every unit increase in number of CVDs care providers. In Afghanistan, Bangladesh, and Nepal, availability of both diagnosis and treatment facilities was associated with increases in readiness scores of 27%, 9%, and 17%, respectively. Additionally, an association was observed between nation-specific facility types and the readiness scores.

**Conclusions:**

Country-specific factors as well as universal factors present in all three nations must be addressed to improve a health facility’s readiness to provide CVDs care. To create focused and efficient country-specific plans to raise the standard of CVD care in South Asia, more investigation is necessary to ascertain the reasons behind country-level variations in the availability of tracer items.

## Introduction

Despite being a global phenomenon, the prevalence of cardiovascular diseases (CVDs) has increased most rapidly over the past 10 years in low- and middle-income countries (LMICs), particularly the highly populous South Asian countries [1]. In the South Asia region—Afghanistan, Bangladesh, and Nepal—CVDs have been gradually rising and are a major cause of all-age death [2]. In Afghanistan, Bangladesh, and Nepal, respectively, CVDs were responsible for 27.8%, 34%, and 24% of all fatalities in 2019 [3].

Surveys that are done at the household level might provide information on indicators like the prevalence of CVDs. These metrics, in turn, are influenced by several variables, including the availability of health services as well as their capacity and level of quality [4]. A health center can function efficiently with the bare minimum of equipment and materials [5]. To gather information about the facilities, supply, and services for sound decision-making, a few chosen indicators must be measured. One way to deal with the expanding CVDs epidemic and aid policymakers in formulating effective, long-term responses is to increase the readiness of health facilities to provide services related to CVDs, including staff and guidelines, equipment and supplies, medicines, and consumables through using the Service Provision Assessment (SPA) tools, which dissect health systems into quantifiable, traceable components.

Since the current healthcare systems were created to provide care primarily for acute infectious diseases, they frequently do not adequately address the rising burden of CVDs in LMICs, particularly South Asia [6]. There were significant shortcomings in the provision of CVD-related services, according to recent assessments of national capacities to manage CVDs in Afghanistan, Bangladesh, and Nepal [7,8,9]. Evidence must be produced to comprehend the gaps in CVD services in those resource-constrained South Asian settings and to investigate workable strategies to enhance the ability of current healthcare systems to deliver CVDs services.

A few studies carried out in several LMICs, including South Asian nations, found low readiness to offer services for noncommunicable diseases like diabetes [10,11,12], hypertension [13,14,15] and chronic respiratory diseases [14,16,17]. There is, however, a dearth of research on CVD readiness and related factors in LMICs [14,17,18,19,20,21,22]. However, most earlier studies on the availability of CVD care in low-resource settings relied on proxy measures made up of generic inputs like the number of healthcare workers and hospital beds [22], or were restricted to local or regional samples [19,20,21]. The results may be skewed because most earlier studies in this area used small sample sizes, unrepresentative samples, or didn’t use a consistent measurement tool to gauge readiness. Furthermore, prior research in this area that centered on national surveys [14,17,18] was primarily done in a single nation. Multicounty studies, however, are required to make a comparative presentation of the facility’s capability to offer CVD services for nations with similar sociocultural aspects.

Based on these considerations, the objectives of this research were to: (1) assess the level of CVD service readiness in three South Asian nations, namely Afghanistan, Bangladesh, and Nepal and (2) identify the variables that influence CVD service readiness in the countries under study.

## Methods

We used ISPOR checklist for retrospective database studies to report our findings [23].

### Data Source

**Figure 1:**
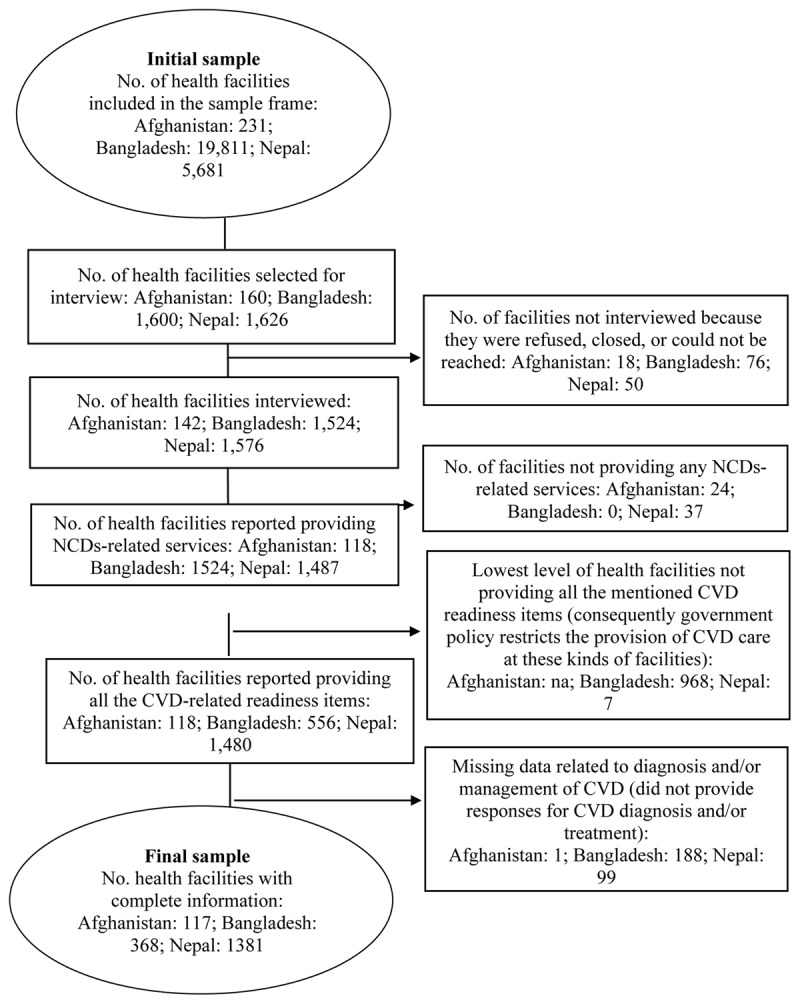
Sample selection of the health facilities: Afghanistan, Bangladesh, and Nepal, SPA surveys.

The present investigation was carried out utilizing secondary data from the cross-sectional, nationally representative Afghanistan Service Provision Assessment Survey 2017–2018 [24], Bangladesh Health Facility Survey 2017 [25], and Nepal Health Facility Survey 2021 [26]. (https://dhsprogramcom/data/Using-DataSets-for-Analysiscfm provides a thorough explanation of how to gain access to and authorization to analyze SPA survey data). The three countries that implemented the SPA survey were chosen after considering the following criteria: i) located within the South Asian region; ii) the presence of CVD-related questions; iii) the availability of specific tracer items for CVDs services; and iv) the most current survey (For details of the sample selection of the facilities included in the SPA sample, see Figure 1).

To get information on health facility availability and readiness in the areas of maternal and child health, family planning, specific noncommunicable diseases, and tuberculosis, the Afghanistan Service Provision Assessment Survey, the Bangladesh Health Facility Survey, and the Nepal Health Facility Survey have been designed [24,25,26]. According to standard operating procedures for healthcare facilities, these surveys assessed the accessibility of staff, basic infrastructure, and logistics, including supplies, necessary drugs, lab services, and infection control procedures.

Four main questionnaires were used in SPA surveys: one for the facility inventory, one for the healthcare provider interview, one for the observation procedures, and one for the exit interviews [24,25,26]. The only data used in this study came from the facility inventory questionnaire. For the facility inventory, interviewers speak with the manager, the person in charge of the facility, or the most senior health worker in charge of client services present at the facility to learn about service readiness, which refers to the availability of amenities, equipment, supplies, laboratory tests, and medications, both generally and for specific services like diabetes, CVDs, and chronic respiratory disease. The questionnaires for the SPA surveys were written in English and were translated into each country’s native language. The basic SPA questions of the Demography and Health Survey Program were fitted and customized to the country’s situation and demands. Countries that used the SPA picked one of two sampling techniques: (1) a census of all facilities in particular districts, which can be used, if desired, for subnational estimates; or (2) a nationally representative simple/systematic random sample of health facilities—to provide national estimates. (For further information on the survey’s details, see S1 Table.)

## Measures

### Outcome

Readiness of a health facility to manage CVDs was the outcome variable in this analysis. This was a composite measure that was generated as a counting score based on the number of vital indicators required for CVDs services within the facility. Three domains were used for evaluating service provision readiness for CVDs, and a total of 10 items/indicators were identified from those three domains: (1) staff and guideline components; (2) equipment and supplies components; and (3) components related to medicine and commodities. The selection of the indicators was determined based on two criteria: (1) they were measured in the SPA survey of the countries under investigation [24,25,26], and (2) complied with the WHO-SARA reference manual [27].

Two indicators were used to evaluate the domain of staff and guidelines: (1) availability of CVD diagnosis and treatment guidelines and (2) at least one staff member delivering the service being trained in CVD diagnosis and treatment in the 24 months prior to the survey. The second domain, components of the equipment and supplies for CVD services was evaluated by means of three indicators: existence of (1) digital blood pressure (BP) machine or manual sphygmomanometer with a stethoscope; (2) adult weighing scale and (3) stethoscope. Medicines and commodities, the third and final domain, was evaluated based on the availability of: (1) aspirin; (2) thiazide; (3) beta blockers (atenolol); (4) calcium channel blockers (amlodipine/nifedipine); and (5) oxygen.

To determine the availability of each of the 10 indicators, binary variables were created, either as presence or absence. A facility was classified as “fully ready” to provide services for CVDs if all these components were present. Additionally, a readiness score was calculated. The readiness score was totaled by adding each indicator’s presence (observed and seen by the interviewers). Each indicator’s contribution to the overall score was given equal weight. The obtained readiness score was calculated as a counting score based on the availability of 10 WHO-SARA indicators [27]. Scores range from 0 to 10.

### Explanatory variables

The explanatory variables were chosen based on two criteria: (1) they were available in the SPA survey of the countries under investigation [24,25,26] and (2) previous research [14,17,18,19,20,21] had established them as important determinants of readiness of health facilities to provide CVD services. The following variables were considered: facility location, managing authority, facility type, external sources of revenue, quality assurance activities, routine management meetings, external supervision, user fees, presence of trained health provider at facility 24 hours with duty schedule or present on-call, feedback on clients’ opinions, health facilities’ ability to perform diagnosis and/or treatment, and number of CVD care providers who had received in-service training in cardiovascular disease services during the 24 months before the survey. The facility’s location was classified as either rural or urban.

To assess managing authority, a dichotomous variable was generated (public: facilities owned by the government, or private: facilities not owned by the government). External revenue sources were classified according to whether the facilities obtained additional (extra) financial assistance from the government, non-government organizations, or none. A dichotomous variable was created to measure quality assurance activities (yes: facility that reported to routinely carry out quality assurance activities, e.g., review of mortality, or audit of registers within the past 12 months, or no: facilities that did not report to routinely carry out quality assurance activities). External supervision was categorized as ‘yes’ for the facility that received supportive supervision from a higher authority such as district or region health management team in the past 12 months; otherwise, the facility was coded as ‘no.’

Routine management meetings were measured using a dichotomous variable (performed: whether the facility reported having regular management meetings at least once every 2–3 months, or not performed). A categorical variable was also created to describe user fees as either none, a separate charge for each service provided to patients, or a fixed fee for all services. The presence of a trained health provider at facility 24 hours with duty schedule or present on-call was categorized as yes versus no. The diagnostic and/or treatment capability of the facility was split into three groups: those with both diagnosis and treatment capacity, those with only diagnosis capacity, and those with only treatment capacity.

Two categories—reviewed and non-reviewed—were created to gather customer feedback. Given the measurement of facility types specific to each country, the following classifications were made: in Afghanistan, national/provincial hospitals, special hospitals, or private hospitals/clinics; in Bangladesh, non-governmental organizations clinics/hospitals, private clinics/hospitals, urban health centers, or district hospitals; and in Nepal, community health units, urban health centers, health posts, primary health care centers, private hospitals, local-level hospitals, or federal/provisional hospitals. The number of CVD providers was treated as a discrete quantitative variable.

## Statistical analyses

First, descriptive analyses were performed to provide general insights into the characteristics of the sample. The mean was utilized in the descriptive analysis to summarize continuous variables. The proportion was used to summarize each categorical variable. Our outcome variable for the investigated countries was a count variable with overdispersion, hence we used a negative binomial regression model to evaluate the effect of each selected explanatory variable on a facility’s readiness to offer CVDs. Utilizing the negative binomial regression models, incidence rate ratios (IRR) were estimated [28]. All explanatory variables were simultaneously included in the multiple regression models. The significance of the association was confirmed using a *P* value of < 0.05 and a 95% confidence interval (CI) for the IRRs. Because the response rate may differ between areas or facility types, and because the facilities sampled in the SPA surveys of the three countries under investigation were not distributed equally (disproportionally), over- and under-sampling was done in the areas with more and fewer facilities. Thus, to restore the true representativeness of the sample data, facility weight was adjusted. We did not combine the data sets in our study; rather, we looked at each one separately; therefore, the weights of the pooled data were not de-normalized. Stata V.16 was used to analyze the data (StataCorp).

## Ethics approval

Since this study used secondary data from a cross-sectional, nationally representative dataset that is accessible online and contains disconnected information for every identity, ethical approval was not required. The US Agency for International Development, Macro International, and the institutional review boards of each nation’s Ministry of Health have given their approval to the SPA survey. All the study procedures were conducted in accordance with the principles of the Declaration of Helsinki as revised in 2013 specimen collection. Prior to taking part in the survey, participants provided their informed consent.

## Results

### Characteristics of surveyed facilities

Table 1 displays the distribution of surveyed facilities in Afghanistan, Bangladesh and Nepal based on their background characteristics. In the three countries surveyed, 89% and 62% of the facilities in Afghanistan and Bangladesh were privately owned, whereas in Nepal only 7.9% of the facilities were publicly owned. Most of the facilities in all the three countries provided both CVDs diagnosis and treatment, ranged from 61.9% in Nepal to 71.5% in Afghanistan. In Afghanistan, Bangladesh, and Nepal, about 54.1%, 61.5%, and 76.9% of facilities reported receiving funds from sources other than the government, such as user fees, faith-based organizations, donor agencies and so on.

**Table 1:** Percentage distribution of surveyed facilities according to background characteristics: Service Provision Assessment Survey, Afghanistan Bangladesh, and Nepal.


VARIABLES	AFGHANISTAN	BANGLADESH	NEPAL

	%	%	%

**Facility location**			

Rural	na	27.1	46.5

Urban		72.9	53.6

**Managing authority**			

Private	89.0	62.0	7.9

Public	11.0	38.0	92.1

**Facility types** *^1^*			

Private hospitals/clinics	87.8		

Special hospitals	7.7	na	na

National/Provincial hospitals	4.4		

**Facility types** *^2^*			

Non-government organization	na	30.0	

clinics/hospitals			na

Private clinics/hospitals		37.8	

Urban health centers		27.9	

District hospitals		4.3	

**Facility types** *^3^*			

Community health units	na	na	7.9

Urban health centers			8.5

Health posts			68.9

Primary health care centers			3.6

Private hospitals			7.9

Local-level hospitals			1.2

Federal/provisional hospitals			1.9

**External sources of revenue**			

None	28.2	3.9	12.1

Other than govt.	17.7	61.5	76.9

Govt.	54.1	34.6	11.1

**Routine quality assurance activities**			

No	73.6	75.3	75.9

Yes	26.4	24.7	24.1

**Routine management meetings**			

Not performed	15.5	na	19.2

Performed	84.5		80.8

**External supervision**			

No	12.3	28.2	4.6

Yes	87.7	71.8	95.4

**User fees**			

None	8.3	8.1	73.1

Separate fees	79.3	84.9	23.5

Fixed for all services	12.4	7.0	3.3

**Presence of health provider, 24 h**			

No	30.8	22.3	58.2

Yes	69.2	77.7	41.8

**Clients’ opinions**			

Not reviewed	60.1	84.3	84.7

Reviewed	39.9	15.7	15.3

**Ability to perform**			

Only diagnosis	3.6	26.1	26.1

Only treat	4.9	3.2	12.0

Both	91.5	70.7	61.9

**No. of CVD care providers**	3.0	3.1	1.8


*Note*: na = data are not available; *^1^*facility type was measured for Afghanistan; *^2^*facility type was measured for Bangladesh: *^3^*facility type was measured for Nepal.

Routine quality assurance procedures were not performed, client opinions were not reviewed in most of the health facilities in all the three countries, and separate patient fees were set for all services in most health facilities in Afghanistan (79.3%) and Bangladesh (84.9%). Bangladesh had a higher average number of CVDs providers (3.1) than Afghanistan (3.0) and Nepal (1.8).

### Availability of CVDs Services

Table 2 shows the distribution of guidelines, equipment, diagnostic instruments, and medicines among surveyed facilities by country. Bangladesh had the highest percentage of CVDs guidelines available (22.4%) in the health facilities evaluated, while Nepal had the lowest (11.3%) and Afghanistan had a higher proportion of trained staff in CVD-related services (21.5%) as compared to Bangladesh (15.3%) and Nepal (12.9%).

**Table 2:** Percentage distribution of surveyed facilities according to availability of guidelines, equipment, and medicines: Service Provision Assessment Survey, Afghanistan Bangladesh, and Nepal.


VARIABLES	AFGHANISTAN	BANGLADESH	NEPAL

	% (95% CI)	% (95% CI)	% (95% CI)

**Staff and guideline**			

Presence of guidelines	20.9 (13.3–29.0)	22.4 (15.5–30.9)	11.3 (9.1–13.8)

Availability of trained staff	21.5 (11.2–37.5)	15.3 (70.5–83.5)	12.9 (10.5–15.7)

**Equipment and supplies**			

BP apparatus	93.4 (78.0–98.3)	97.1 (93.5–98.7)	97.4 (95.7–98.5)

Adult scale	70.5 (58.4–80.2)	84.0 (78.4–88.4)	95.1 (93.2–96.5)

Stethoscope	93.8 (77.8–98.5)	97.6 (93.7–99.1)	98.4 (96.9–99.2)

**Medicines and commodities**			

Aspirin	83.9 (68.9–92.4)	33.1 (26.5–40.4)	17.7 (15.3–20.4)

Thiazide	35.7 (23.7–49.8)	16.4 (11.5–22.9)	6.7 (5.5–8.3)

Beta blockers (atenolol)	84.1 (69.1–92.6)	36.6 (29.9–43.7)	32.4 (28.9–36.1)

Calcium channel blockers	89.0 (76.5–95.2)	47.3 (40.2–54.6)	61.5 (57.7–65.4)

(amlodipine/nifedipine)			

Oxygen	77.0 (67.7–84.3)	55.3 (48.1–62.3)	27.6 (24.4–31.1)

**Fully ready**	0.5 (0.07–4.0)	0.03 (0.01–0.08)	0.09 (0.04–0.24)

**Readiness score**	6.7 (6.2–7.2)	5.6 (5.3–5.9)	4.6 (4.51–4.71)


*Note*: CI = Confidence interval.

The equipment in the facilities in Afghanistan, Bangladesh, and Nepal was as follows: 93.8%, 97.6%, and 98.4% had stethoscopes; 93.4%, 97.1%, and 97.4% had blood pressure monitors; and 70.5%, 84%, and 95.1% had adult weighing scales.

In all three of the study countries, the most readily available medications were aspirin, beta blockers, and calcium channel blockers (amlodipine/nifedipine). Aspirin, beta blockers, calcium channel blockers (amlodipine/nifedipine), and thiazide were all more common in Afghanistan (83.9%, 84.1%, 89.0%, and 35.7%) than in Bangladesh (33.1%, 36.6%, 47.3%, and 16.4%) and Nepal (17.7%, 32.4%, 61.5%, and 6.7%). Compared to 55.3% in Bangladesh and 27.6% in Nepal, 77% of facilities in Afghanistan had oxygen in either cylinder, concentrators, or an oxygen distribution system. The overall mean readiness scores were higher in Afghanistan (6.7), followed by Bangladesh (5.6) and Nepal (4.6). The results also indicate that in all the health facilities examined in the three countries, only 0.5% of facilities in Afghanistan, 0.03% of facilities in Bangladesh, and 0.09% of facilities in Nepal had all 10 important items for CVD management.

### Factors associated with readiness to manage CVDs

The results of the negative binomial regression model analysis for variables related to health facility readiness to manage CVDs in the countries under study are shown in Table 3. Provided that the other variables in the model remain unchanged, the readiness score for Afghanistan and Nepal is expected to fall by 59% (IRR 0.59; 95% CI 0.46 to 0.76) and 73% (IRR 0.73; 95% CI 0.66 to 0.81) for public managing authority versus private.

**Table 3:** Models of negative binomial regression for variables associated with health facility readiness to manage CVDs: Service Provision Assessment Survey, Afghanistan Bangladesh, and Nepal.


VARIABLES	AFGHANISTAN	BANGLADESH	NEPAL

	IRR (95% CI)	IRR (95% CI)	IRR (95% CI)

**Facility location**			

Rural	na	1.00	1.00

Urban		1.05 (0.97–1.13)	0.98 (0.93–1.03)

**Managing authority**			

Private	1.00	1.00	1.00

Public	0.59 (0.46–0.76)*^a^*	1.05 (0.91–1.21)	0.73 (0.66–0.81)*^a^*

**Facility types** *^1^*			

Private hospitals/clinics	1.00	na	na

Special hospitals	1.42 (1.17–1.72)*^b^*		

National/Provincial hospitals	1.65 (1.16–2.34)*^b^*		

**Facility types** *^2^*			

Non-government organization	na	1.00	na

clinics/hospitals			

Private clinics/hospitals		0.91 (0.80–1.04)	

Urban health centers		0.62 (0.46–0.85)*^b^*	

District hospitals		0.89 (0.51–0.93)	

**Facility types** *^3^*			

Community health units	na	na	1.00

Urban health centers			1.02 (0.97–1.09)

Health posts			1.04 0.95–1.19)

Primary health centers			1.31 (1.21–1.42)*^a^*

Private hospitals			–

Local-level hospitals			1.42 (1.28–1.57)*^a^*

Federal/provisional hospitals			1.33 (1.20–1.48)*^a^*

**External sources of revenue**			

Government	1.00	1.00	1.00

Other than government	1.01 (0.90–1.13)	1.05 (0.68–1.93)	1.03 (0.97–1.11)

None	0.77 (0.64–0.92)*^b^*	0.79 (0.68–0.97)*^c^*	1.08 (0.99–1.19)

**Routine quality assurance activities**			

No	1.00	1.00	1.00

Yes	0.99 (0.92–1.06)	1.05 (0.95–1.16)	1.06 (1.01–1.11)*^c^*

**Routine management meetings**			

Not performed	1.00	na	1.00

Performed	0.97 (0.83–1.13)		1.01 (0.96–1.06)

**External supervision**			

No	1.00	1.00	1.00

Yes	1.47 (0.96–2.26)	0.98 (0.86–1.11)	1.03 (0.95–1.14)

**User fees**			

None	1.00	1.00	1.00

Separate fees	0.80 (0.59–1.10)	1.02 (0.92–1.14)	1.10 (1.04–1.18)*^b^*

Fixed for all services	1.01 (0.72–1.41)	0.97 (0.84–1.13)	1.04 (0.95–1.14)

**Presence of health provider, 24 h**			

No	1.00	1.00	1.00

Yes	1.16 (1.06–1.28)*^b^*	1.19 (1.08–1.33)*^b^*	1.02 (0.97–1.07)

**Clients’ opinions**			

Not reviewed	1.00	1.00	1.00

Reviewed	1.09 (1.01–1.17)*^c^*	1.09 (1.02–1.19)*^c^*	1.04 (0.99–1.09)

**Ability to perform**			

Only diagnosis	1.00	1.00	1.00

Only treat	1.16 (0.97–1.38)	1.00 (0.91–1.17)	1.16 (1.08–1.26)*^a^*

Both	1.27 (1.05–1.53)*^c^*	1.09 (1.01–1.17)*^c^*	1.17 (1.09–1.25)*^a^*

**No. of CVD care providers**	1.02 (1.01–1.04)*^b^*	1.02 (1.01–1.04)*^b^*	1.02 (1.01–1.04)*^a^*


*Note*: CI = confidence interval; IRR = incidence risk ratio; na = data were not available; *^1^*facility type was measured for Afghanistan; *^2^*facility type was measured for Bangladesh; *^3^*facility type was measured for Nepal. Here a, b, and c indicate *p* < 0.001, *p* < 0.01 and *p* < 0.05.

Compared to private hospitals/clinics in Afghanistan, special hospitals and national/provisional hospitals were associated with increases in readiness scores of 42%, and 65%, respectively. In Bangladesh, the readiness score is expected to decrease by 38% for urban health centers versus non-government organization clinics/hospitals. On the other hand, primary health care centers, local-level hospitals, and federal/provisional hospitals in Nepal were associated with 31%, 42%, and 33% increases in readiness score, respectively, as compared to community heath units.

When compared with their counterparts, the presence of trained healthcare providers and when the clients’ opinions were reviewed were associated with a respective 16% and 9% increase in readiness score in Afghanistan. In Nepal, when separate fees were set for all services, there was an associated 10% increase in readiness score as compared to when no fees were set. In Bangladesh, the presence of trained healthcare providers was associated with a 19% increase in readiness score.

In all three countries, an increase in the number of CVD providers was associated with an increase in readiness scores, i.e., we ought to expect a 0.02 unit rise in the readiness scores for the three nations under study for every unit increase in the number of CVD care providers. In Afghanistan, Bangladesh and Nepal, facilities with both diagnostic and treatment capacity were linked to 27%, 9%, and 17% increase in readiness scores, respectively.

## Discussion

### Major findings

This is the first multi-country study to evaluate the state of CVD service readiness in three low-resource South Asian nations: Afghanistan, Bangladesh, and Nepal, as well as the variables affecting CVD service readiness in those countries. There are four main findings: (1) among the 10 important components for CVDs management, relatively low mean readiness scores were found in all three countries studied, ranging from 6.7 in Afghanistan to 4.6 in Nepal; (2) all three countries lack common access to staff availability and the presence of guidelines services, except for supplies and equipment; (3) lower levels of medicine and other commodities were seen in Bangladesh and Nepal, with Afghanistan being the exception; and (4) although the factors linked to increased readiness scores varied by country, the facility types, increases in the number of CVD care providers, and the ability to perform both diagnosis and treatment are similar factors linked to increased readiness scores in all three countries.

### Comparable to other studies

The observation of a low level of CVD care facility readiness in the three countries under investigation shows that health systems in those low-resource settings have significant challenges in providing CVD care services. Previous small-scale investigations in Afghanistan [29], Bangladesh [30], and Nepal [31] found that health facilities weren’t ready to provide CVD-related services. Other LMICs [19,20,21] have found that they are facing similar difficulties. Health facilities’ lack of readiness in all three countries may be due to inadequate funding raised through domestic and international channels and a lack of political importance [32] given to combating non-communicable disease. These findings suggest that health facilities in these resource-constrained nations should be better prepared to offer CVD services to battle the rising CVD epidemic.

In providing CVD care, having a trained and competent team and having CVD guidelines are important factors [33]. Our research showed that, in all three of the countries that we looked at, the availability of trained staff and guidance services is far from universal. This discovery is particularly important since it can point to the existence of access restrictions for patients who are more vulnerable. These results are consistent with earlier evaluations of CVD care [14,17,18,19,20,21] in other LMICs.

Given the high burden of communicable diseases in LMICs, non-communicable diseases are given less priority when allocating national resources for the health care system [34], which is why Afghanistan, Bangladesh, and Nepal lack training facilities for CVD care. Moreover, it takes time to train specialists in cardiovascular care. Therefore, it is reasonable that in the near future these countries may not be able to produce enough physicians with the appropriate training in CVD care to meet the needs of the population about CVDs. The transfer of healthcare obligations [35] normally performed by doctors to other healthcare professionals—such as community health workers, nurses, and pharmacists—as part of team-based care is known as task-shifting. These professionals can be trained to manage CVDs, which could be a useful way to address the current shortages in those countries’ health workforces for CVD care. In LMICs, task-shifting has been successfully implemented for cardiovascular care [36,37,38] and is acknowledged as a valuable paradigm for HIV, maternity, and childcare [39].

Only Afghanistan meets the WHO’s proposed 80% availability goal for key medicines for CVD care [40], except for thiazide; the current study found that all essential CVD medications were considerably less readily available in healthcare facilities in Bangladesh and Nepal. This demonstrates how slowly these countries are addressing the rising CVD burden. In the treatment of patients with heart illness, oxygen supplementation has long been a standard [41]. According to the current study, Bangladesh and Nepal are seeing worsening oxygen availability, with all three countries falling short of the WHO’s planned 80% availability goal. Therefore, it is crucial that the government should take the required actions to guarantee that there is access to life-saving oxygen therapy for CVD care.

Achieving a balance between human and physical resources and guaranteeing the system’s efficacy depend on maintaining an adequate number of health professionals [42,43]. The relationship between an increase in the number of CVDs care providers and an increase in the capability of healthcare facilities to deliver CVDs services is consistent across the three countries under study. This is because when healthcare consumables, such as drugs, supplies, and diagnostic tools are easily accessible, it may affect the capacity of healthcare systems to attract and retain specialists in their fields to maintain the system’s functionality [42,43].

According to the current study, privately managed facilities in Afghanistan and Nepal had higher readiness scores than those under public management. These results are consistent with those of earlier research projects [14,17,44] carried out in low-resource environments. One theory is that because privately held hospitals are run for profit, their management teams are more responsible and patient-focused, and as a result, they tend to offer higher-quality services to attract more customers and make a profit. As a result, the appropriate authorities need to focus more on the low level of readiness in the public sector.

Specialist hospitals and national/provincial hospitals in Afghanistan are more likely to be ready to provide CVDs services than private hospitals/clinics. This is plausible because the government predominantly funds specialist hospitals and national/provincial hospitals in Afghanistan, and CVDs care is primarily given in those hospitals [45].

The readiness scores for providing CVD-related services in urban health centers in Bangladesh was rather low. Urban health centers act as a hub for primary healthcare facilities that primarily serve the urban poor seeking treatment for non-communicable diseases [46]. Though Bangladesh’s government established non-communicable diseases corners in urban health centers, the findings suggest that Bangladesh’s primary healthcare systems are still lacking in their ability to combat CVDs [46]. This is in direct opposition to WHO efforts to prioritize the introduction of Package for Essential urban health centers strategies in primary healthcare facilities in low-resource settings for the management of non-communicable diseases [47]. The readiness score was found to be low in community health units in Nepal as compared to primary health centers, local-level hospitals, and federal/provisional hospitals. In Nepal, community health units are regarded as the first rung of the health care system and are primarily in charge of providing services related to family planning and maternity and child health [48]. Therefore, compared to the second (primary healthcare centers) and third tiers of the health system (local-level hospitals and federal/provisional hospitals), the low readiness of community health units is plausible.

The findings showed that higher readiness scores in Afghanistan was also connected to the government’s external sources of funding. This result is comparable with those of a multicounty study [49], which discovered a link between obtaining funding from outside organizations and the general readiness of healthcare institutions.

A key component of staff management in a healthcare facility is staff scheduling, which is the process of creating duty schedules for its workers [50]. According to our data, in Afghanistan and Bangladesh, facilities with 24-hour personnel scheduling had a higher likelihood of having higher readiness scores than those without. Facilities with round-the-clock staffing may be more likely to offer top-notch services and better cater to patients’ demands. These facilities are therefore more likely to boost service availability, possibly leading to high readiness scores.

According to the results of this study, the ability of health facilities in the three nations under investigation to perform both diagnosis and treatment was more likely to be correlated with higher readiness scores. These results are congruent with those of other research investigations [10,49,51] carried out in various other developing countries. Another significant discovery is that many health facilities in Bangladesh and Nepal lack treatment facilities, despite having ample diagnostic capabilities. After conducting additional analysis, we found that most of these facilities are primary healthcare systems, which also have a lower percentage of trained CVD providers than other kinds of health facilities. Even though diagnostic capabilities are available, the lack of CVD providers may be the reason for the absence of treatment related to CVD, or it may be because government policy in those nations forbids the provision of CVD care at these kinds of facilities.

The payment for health services by consumers is a policy option that many developing nations have used to raise money to meet the expanding demand for health care services [43]. According to this study, we found that in Nepal, health facilities those charged separate user fees or expenses for client services were more likely to have higher readiness score.

For the system to function well and to strike a balance between the use of physical and human resources, it is essential to maintain enough health professionals [42]. The relationship between an increase in the number of CVDs care providers and an increase in the capability of healthcare facilities to deliver CVDs services is consistent across the three countries under study. This is because when healthcare consumables, such as drugs, supplies, and diagnostic tools, are easily accessible [5], it may affect the capacity of healthcare systems to attract and retain specialists in their fields to maintain the system’s functionality.

‘Quality assurance’ is the process of assuring and upholding a high standard of service within healthcare facilities [52]. The findings of this study indicated that in Nepal, facilities that engaged in routine quality assurance activities had a higher likelihood of achieving higher readiness scores. This is because quality assurance calls for ongoing evaluation and monitoring to enhance service delivery.

Additionally, according to our data, in Afghanistan and Bangladesh, facilities that have a system in place for gathering feedback from clients tend to have higher readiness scores than those that didn’t. The greater readiness score among facilities that have a client feedback system demonstrates the importance of patient experiences and feedback for service improvement [52,53,54].

### Strength and limitations

This study offers several features. First, the most recent nationally representative sample of public and commercial health facilities from three countries in the South Asian region, containing a significant number of health facilities (Afghanistan: n = 117; Bangladesh: n = 368; and Nepal: n = 1,381), was used to examine the data. Second, the SPA uses thorough interviewer training, standardized measuring methods and methodologies, the same core questionnaire, as well as pretesting tools, to achieve standardization and comparability across all sites and time periods. Third, to reflect the clinical reality of the research, the outcome variables were created using measurements from the WHO-SARA guideline. Lastly, trained survey enumerators investigated healthcare facilities, noting the availability of staff, guidelines, supplies and equipment, and medications.

Our study has some significant shortcomings. First, while CVDs include a range of conditions affecting the heart and blood vessels, including rheumatic heart disease, congenital heart disease, coronary heart disease, peripheral arterial disease, deep vein thrombosis, and pulmonary embolism, SPA surveys did not gather data regarding individual CVD disorders. Additionally, the index’s consumables are a mishmash of items for various ailments, such as oxygen for acute myocardial infraction and/or heart failure, beta blockers and aspirin for chronic ischemic heart disease, and calcium channel blockers and thiazides for hypertension. Furthermore, the listed medications and equipment only provide a partial picture of the resources required for a given form of CVD. For instance, ACEi and ARB are excluded even though they are among the most used medications for treating hypertension and are necessary for left ventricular failure. Additionally, absent are two crucial medications for heart failure: aldosterone antagonists and loop diuretics. In terms of primary and secondary prevention, neither are statins. As a result, whereas drugs are tracers for CVD in our study, this does not indicate that CVD care is ready or that it can be effectively managed. Second, the SPA surveys did not specify the kinds of CVDs being diagnosed and treated at these facilities, even though we had taken into consideration CVD diagnosis and treatment guidelines as one of the tracer items for the readiness score. These points should eventually be considered in further SPA surveys so that data for specific CVDs in specific countries can be directly compared for research purposes.

Third, the cross-sectional design of this study offers just a snapshot of the circumstances and lacks long-term proof of the accessibility and readiness of health facilities to provide services for CVDs. Fourth, our findings cannot be generalized to other South Asian nations due to the unavailability of SPA surveys in those countries. Fifth, while this study examined several areas, such as the availability of drugs, equipment, and guidelines, most of these were very basic assessments, and many other instruments and equipment that are essential to the management of CVDs, such as electrocardiograms and other technologies, were not considered.

Sixth, even though tertiary-level health facilities offer services linked to CVD, no data from these types of institutions was included in the Bangladesh Health Facility Survey 2017. To help government decision-makers make informed decisions, this should be considered in future research. Finally, as there is no research on the relative weights of the WHO-SARA instrument’s CVDs tracer items, we applied equal weights for all items in the CVD-specific readiness scores. It is possible that this overestimated or understated the degree of CVDs service readiness.

## Conclusions

In three countries with high CVD prevalence, we found that health facilities lack the capacity to offer services for the disease. Afghanistan received a mean readiness score of 6.7, Bangladesh a score of 5.6, and Nepal a score of 4.6. Except for equipment and supplies, none of the three countries studied have a universal availability of staff and guidance services. Afghanistan has the highest levels of medicine and other commodities availability, with Bangladesh and Nepal having the lower levels. Facility types, increases in the availability of CVDs care providers, and the capacity to execute both diagnosis and treatment are similar factors associated to increasing readiness scores in all three nations, even though the factors varied by country. To increase a health facility’s readiness to deliver CVDs care, both country-specific and universal factors that exist in all three countries must be addressed.

## Data Accessibility Statement

The data are available in a public, open access repository at https://dhsprogram.com/data/AccessInstructions.cfm.

## Additional File

The additional file for this article can be found as follows:

10.5334/gh.1311.s1 S1 Table. Survey details of Afghanistan Service Provision Assessment Survey 2018–19, Bangladesh Health Facility Survey 2017, and Nepal Health Facility Survey 2021.
