# Supplementary material for: Health Facilities Readiness and Determinants to Manage Cardiovascular Disease in Afghanistan, Bangladesh, and Nepal: Evidence from the National Service Provision Assessment Survey
Source: Glob Heart. 2024 Mar 20;19(1):31. doi: 10.5334/gh.1311 (PMC10959132; doi:10.5334/gh.1311)
Supplement: S1 Table. — Survey details of Afghanistan Service Provision Assessment Survey 2018–19, Bangladesh Health Facility Survey 2017, and Nepal Health Facility Survey 2021. [file gh-19-1-1311-s1.pdf]

S1 Table Survey details of Afghanistan Service Provision Assessment Survey 2018-19, Bangladesh Health Facility Survey 2017, and Nepal Health Facility Survey 2021

Country	Survey year	Name of the survey	Facilities selected for the survey sample
Afghanistan	2018–19	Afghanistan Service Provision Assessment survey	Census of public hospitals. private hospitals and NGO/private clinics in six provinces namely Nangarhar, Paktya, Kunduz, Balkh, Kandahar, and Herat. In Kabul province there was a census of public hospitals. private hospitals and random selection of NGO/private clinics
Bangladesh	2017	Bangladesh Health Facility Survey	Combining a census of public hospitals (district hospitals and mother and child welfare centers) with random sampling of other public hospitals, private hospitals, and NGO/private clinics
Nepal	2021	Nepal Health Facility Survey	Census of public hospitals, private hospitals, and NGO/private clinics
